# Investigation of *Blastocystis spp*. in patients with inflammatory bowel disease by direct microscopy and molecular methods

**DOI:** 10.4314/ahs.v25i1.8

**Published:** 2025-03

**Authors:** Şentürkoğlu Feride, Samadzade Rugıyya, Korkmaz Hüseyin, Maçin Salih

**Affiliations:** 1 Selçuk University, Faculty of Medicine, Department of Medical Microbiology, Konya, Turkey; 2 Selçuk University, Faculty of Medicine, Department of Internal Medicine, Konya, Turkey

**Keywords:** *Blastocystis spp*, Crohn's Disease, Ulcerative Colitis

## Abstract

**Background:**

*Blastocystis spp*. is a single-celled, eukaryotic protozoan parasite that lives in the intestinal tract of human and animal hosts.

**Objectives:**

The aim of this study is to investigate the relationship between Inflammatory Bowel Diseases and *Blastocystis spp*.

**Methods:**

Crohn's disease (n:25), Ulcerative Colitis patients (n:30) and healthy individuals (n:30) included into the study. Stool samples were investigated by direct microscopic examination and real time PCR method.

**Results:**

*Blastocystis spp*. was detected in five (16.6%) of Ulcerative Colitis, one (4%) of Crohn's disease, and one (3.3%) of the healthy control group by direct microscopy. *Blastocystis spp*. DNA was detected in seven (23.3%) of Ulcerative Colitis, one (4%) of Crohn's disease and one (3.3%) of the healthy control group by real time PCR method.

**Conclusions:**

The prevalence of *Blastocystis spp*. was found to be higher in Ulcerative Colitis patients (23.3%) than in Crohn's disease (4%) and healthy controls (3.3%). According to these results, it has been shown that there may be a possible relationship between Ulcerative Colitis and *Blastocystis spp*.

## Introduction

Inflammatory Bowel Disease (IBD) is a chronic disease that affects many people worldwide, grouped in two different clinical forms as Crohn's Disease (CD) and Ulcerative Colitis (UC). These diseases are characterized by severe attacks of intestinal inflammation followed by a period of remission, which greatly reduces the quality of life of the patients^1^. The pathogenesis of IBD is considered to be a result of an interplay between genetic susceptibility and environmental risk factors, including smoking, unfavourable diet and lifestyle, which subsequently lead to an inappropriate intestinal immune activation and a proin-flammatory intestinal microbiome^2^,^3^.

Monitoring the disease activity should be based on a combination of clinical symptoms and findings of laboratory investigations, colonoscopy, and imaging examinations^4^. Colonoscopy remains the reference standard method for assessment of the activity and extent of IBD, which involves both the colonic and rectal mucosa. There is extensive evidence shows the importance of both mucosal healing and histological healing and their excellent correlation with reduced risk of relapse and hospitalization^5^,^6^.

*Blastocystis spp*. is a unicellular, anaerobic, eukaryotic protozoan parasite that lives in the intestines of humans and other hosts (birds and other mammals)^7^. There are four main morphological forms: “vacuolar”, “granular”, “amoeboid” and “cyst”^8^. It is still controversial whether Blastocystis spp. is pathogenic or pathogenic^9^,^10^.

Intestinal inflammation and edema have been reported in individuals infected with *Blastocystis spp*. In an animal model, extensive infiltration of inflammatory cells into the colon and inflammation with edematous lamina propria in the cecum and colon have been reported following *Blastocystis spp*. infection in histopathological examinations^11^. In the presence of the parasite; some gastrointestinal symptoms such as “diarrhea”, “abdominal pain”, “vomiting”, “constipation” and “gas” can be seen. It is thought that *Blastocystis spp*. may play a role in the formation of some chronic gastrointestinal diseases such as Irritable Bowel Syndrome (IBS) and IBD^12^.

The aim of this study is to investigate the presence of Blastocystis spp. in patients diagnosed with IBD and to determine whether there is a significant relationship between IBD and Blastocystis spp. In addition, it is aimed to compare the results of direct microscopic examination (DM) and molecular method (polymerase chain reaction; PCR) used in the laboratory diagnosis of *Blastocystis spp*.

## Methods

### Selection of Study Groups, Collection and Storage of Samples

A total of 85 individuals, including 25 patients with CD, 30 patients with UC, and 30 healthy controls without IBD, who were followed up in the Gastroenterology outpatient clinic of Selcuk University Medical Faculty Hospital, were included in the study. Stool samples sent in clean, plastic stool containers that do not contain fixatives and were examined without delay. The samples were placed in two eppendorphes and placed in a -80 °C freezer for future molecular study.

### Examination of Stool Samples by Direct Microscopy Method

The presence of *Blastocystis spp*. forms in 85 stool samples was investigated by the direct microscopy method within the scope of routine parasitological examination. Stool samples were carefully mixed with saline (0.9% NaCl) and Lugol's solution dropped on both ends of the slide and examined under the light microscope at 20X and 40X magnification.

### Genomic DNA Isolation from Stool Samples

DNA Isolation was performed using the ZymoBIOMICSTM DNA Miniprep Kit Protocol (Merck KGaA, Darmstadt, Germany). These isolates obtained were placed in small eppendorf tubes numbered according to the names of the volunteers and placed in the freezer at a temperature of -20°C.

### Real-time Polymerase Chain Reaction

The isolates obtained by DNA isolation process, “abm BlasTaq 2X qPCR MasterMix” ready kit (Thermo Fisher Scientific, Germany), Fluorogenic SYBR Green (Thermo Fisher Scientific, Germany), 96 Plate with appropriate primers and Roche Light Cycler 96 device (Basel, Switzerland) were used as mix. In accordance with the study protocol, the mix mixture, primers and the isolate matching the well number were placed in each well of the 96well plate and the amplification process was started. The 96 plate, covered with a sealer, was loaded into the Roche LightCycler 96 device. Real-Time PCR was started in the appropriate temperature protocol and performed in 40 cycles.

### Statistical analysis

All statistical analyzes were performed with R version 3.6.0 (The R Foundation for Statistical Computing, Veinna, Austria; https://www.r-project.org). The normality of the data was checked with the help of the Shapiro-Wilk test of normality and the Q-Q graph, and the homogeneity of the variances was checked with the Levene test. Descriptive statistics for numerical data were presented as mean ± standard deviation (minimum – maximum), while descriptive statistics for categorical variables were presented as numbers (n) and percentage (%). Statistical diagnostic measures were given with a 95% confidence interval. The significance level was taken as 5% in the evaluation of the hypotheses.

## Ethical statement

Our study was accepted as a research project with the ethics committee decision dated 30.09.2020 and numbered 2020/427 of the Selcuk University Faculty of Medicine Local Ethics Committee.

## Results

Individuals aged between 18 - 71 (42.07 ± 14.15), and 49 (57.6%) of all were men (Table 2).

**Table 2:** Demographic characteristics by patient groups

Groups	Age	Gender
	
	Medium ± SS (min – max)	*n* (%) Male / Famale
Ulcerative Colitis patient group (*n*=30)	44.07 ± 13.66 (19–71)	18(60)/12 (40)
Crohn's disease patient group (*n*=25)	41.68 ± 15.13 (18 – 69)	16 (64) / 9 (36)
Healthy Control group (*n*=30)	40.40 ± 14.04 (19–71)	15 (50) / 15 (50)
*p*-value	.602^1^	.549^2^

Data were presented as mean ± standard deviation (SD) or frequency (n) and percentile (%).

1 One-way analysis of variance

2 Pearson chi-square test

*Blastocystis spp*. was found by direct microscopy in 5 (16.6%) stool samples from 30 UC patients and in 1 (4%) of 25 CD samples. In the healthy control group, *Blastocystis spp*. was detected in 1 (3.3%) of 30 samples. With real-time PCR, 7 (23.3%) samples were found to belong to the UC, and 1 (4%) to the CD group. *Blastocystis spp*. DNA was detected in (3.3%) of the samples belonging to the control group. *Blastocystis spp*. prevalence in UC controls (3.3%) (Table 2). There was no difference between CD and control groups.

*Blastocystis spp*. was detected in 9 (10.6%) out of 85 individuals by PCR method. *Blastocystis* spp. was observed in 7 (8.2%) of these 85 individuals with direct microscopy (Table 3). Of the 9 Blastocystis spp. detected by PCR, 7 (sensitivity =77.8, 95% CI=40-97.2) were also detected by direct microscopy. Negative results with PCR, were also negative (specificity=100, 95% CI=95.3-100) by direct microscopy. The corelation between PCR and direct microscopy findings was 86.2% (ϰ=0.862).

**Table 3:** Detection rates of *Blastocystis* spp. according to groups by real-time PCR method

Groups	*Blastocystis* spp.

	*n* (%)
Ulcerative Colitis patients (*n*=30)	7 (23.3)
Crohn's Disease patients (*n*=25)	1 (4)
Healthy Control group (*n*=30)	1 (3.3)
*p*-value	.036^1^

Data were presented as mean ± standard deviation (SD) or frequency (n) and percentile (%)

1 Fisher-Freeman-Halton test

## Discussion

*Blastocystis spp.*, is thought to be associated with IBD, however it can be detected from asymptomatic individuals. Studies have shown that intestinal inflammation caused by the parasites may cause specific inflammatory changes in the intestinal wall. In an in vivo study with mice, the parasite was reported to cause inflammatory cell infiltration and active colitis in the large intestine of infected mice^13^,^14^. In addition, in the ultrastructural examination of the effect of *Blastocystis* spp. on the intestinal mucosa in mice, it was found that the parasite invades the fold of microvilli on the surface of the intestinal cavity and ileocecum mucosa, where it is partially destruction has been observed15. In a similar study, inflammatory cell aggregations were observed in the histopathological examination of the cecum. This is because of the ability of Blastocystis spp. to stimulate the release of chemokines and cytokines such as interleukin (IL)-8, granulocyte/macrophage colony stimulating factor^16^.

The presence of *Blastocystis* spp. was examined by direct microscopy, Trichrome staining and culture methods in 150 IBD and 160 healthy control groups^17^. *Blastocystis* spp. was found to be positive in 50 patients (33.3%) and18 (12%) in the control group. The results showed a possible association between IBD and *Blastocystis* spp. infection. Consistent with this study, the prevalence of *Blastocystis* spp. in patients with IBD was also higher in our study.

Many studies have shown that *Blastocystis* spp. has been shown to be associated with intestinal diseases. In China, Tai et al. found the presence of *Blastocystis* spp. in 6 of 49 patients with persistently recurrent symptomatic UC as a result of microscopic stool examination^18^. These patients appeared to have fully recovered after 14 days of metronidazole treatment. These results indicate that Blastocystis spp. may have a role in the pathogenesis of UC.

Obligate anaerobic bacterial communities predominate and the environment is low in oxygen in the healthy gut. In patients suffering from inflammatory bowel diseases, it is assumed that there is a deterioration in anaerobiosis as a result of the shift of the gut microbiota from obligate anaerobes to facultative anaerobes^19^. They emphasized that *Blastocystis* spp. is also a strict anaerobic parasite and is dependent on other components of the microbiota in order to maintain its existence in the human intestine^20^.

The most common diagnostic technique used worldwide for the diagnosis of *Blastocystis* spp. is direct microscopic examination. Molecular methods are not yet used in routine diagnosis due to reasons such as cost and need for experienced personnel. However, in recent years, molecular tests have been found to be highly sensitive and specific in detecting *Blastocystis* spp^21^. Due to the wide genotypic diversity of Blastocystis spp., the selection of primers in PCR becomes diagnostically important. Some primers can only amplify certain subtypes, which may cause some subtypes to be missed in the analysis^22^,^23^.

Boral et al. included a total of 60 IBD patients (30 with UC and 30 with CD) and 40 healthy control groups in their study^24^. Stool samples collected from this study group were by microscopy, culture, ELISA and PCR methods and *Blastocystis* spp. existence was investigated. In microscopic examination, *Blastocystis* spp. was found in 4 (13.3%) stool samples of 30 UC patients and 2 (6.6%) of 30 CD samples. In the healthy control group samples, Blastocystis spp. was observed in 2 (5%) of 40 samples.

By PCR, *Blastocystis* spp. was detected in 6 (20%) stool samples of 30 UC patients and 2 (6.6%) of 30 CD samples. In 6 (15%) of the control group samples, *Blastocystis* spp. DNA has been detected. While all of the samples detected to have *Blastocystis* spp. by microscopy and culture were positive by ELISA and PCR methods, some of the samples found to be positive by ELISA or PCR were observed to be negative by microscopy and culture. As a result of these data, they interpreted the sensitivity of PCR as high.

Poirier et al. compared the real-time PCR method, with direct microscopy and xenic in vitro stool culture analysis in 94 immunocompromised patients with hematological malignancies^25^. They detected 8 Blastocystis spp. positive samples with DM, 14 Blastocystis spp. by XIVC and 27 *Blastocystis* spp. positive samples by real-time PCR. Dagci et al. the presence of *Blastocystis* spp. in 617 patients included in the study was investigated by three different methods^24^. *Blastocystis* spp. was detected in 11 (1.7%) samples in direct microscopy, 80 (12.9%) in culture, and 94 (15.2%) in PCR. When comparing the three diagnostic methods, real-time PCR was reported to be the most sensitive and specific method.

Roberts et al. analyzed the stools of 513 patients with permanent modified iron-hematoxylin dye and detected *Blastocystis* spp. in 47 (9%) samples26. When B11400ForC and b11710RevC were used as primers in PCR only one Blastocystis spp. was positive in 65 (12%) samples. F1 primers specific for SSU rDNA of *Blastocystis* spp. and BHCRseq3 primers were used in PCR 2. *Blastocystis* spp. It was found positive in 92 (18%) samples. DNA sequencing was performed for confirmation on all PCR positive samples. As a result of the study, it was emphasized that the most sensitive method for detecting *Blastocystis* spp. was PCR, and the sensitivity of the microscopic diagnosis method was low. Consistent with other studies PCR was found to be the most sensitive method in our study. According to the results, the prevalence of *Blastocystis* spp. in patients with UC (23.3%) was significantly higher than in patients with CD (4%) and healthy control groups (3.3%) (p= 0361). In pathogenicity studies, *Blastocystis* spp. and factors originating from the host should be considered collectively. In our study, a possible relationship between UC and *Blastocystis* spp. was observed. However, no significant difference was found between the CD group and the control group in terms of the presence of *Blastocystis* spp.

## Figures and Tables

**Table 1:** Primer sequences obtained from the *Blastocystis* spp. SSU rRNA gene region

Primer	Primer Sequence (5′ – 3′)	Base Pair (bp)
**BL18SPPF1 (forward)**	**(5′** – AGTAGTCATACGCTCGTCTCAAA – **3′)**	320
**BL18SR2PP (reverse)**	**(5′** – TCTTCGTTACCCGTTACTGC – **3′)**	342

**Table 4:** Diagnostic performance of Direct Microscopy

	PCR results		Total

	Negative	Positive	
DM results			
Negative	76 (True Negative–TN)	2 (False Negative–FN)	78 (91.8%)
Positive	0 (False Positive–FP)	7 (True Positive–TP)	7 (8.2%)
Total	76 (89.4%)	9 (10.6%)	85
Statistical Diagnostic Measures			
Sensitivity (%95 CI)	77.8 (40 – 97.2)		
Specificity (%95 CI)	100 (95.3 – 100)		
PPV (%95 CI)	100 (59 – 100)		
NPV (%95 CI)	97.4 (91 – 99.7)		
Accuracy (%95 CI)	97.6 (91.8 – 99.7)		
*K*	0.862		

**TN:** true negative, **FN:** false negative, **FP:** false positive, **TP:** true positive, 95% **CI:** 95% confidence interval, Sensitivity: sensitivity, Specificity: specificity, **PPV:** positive predictive value, **NPV:** negative predictive value, **Accuracy:** accuracy, **κ:** Value of Kappa test statistic
